# Eosinophilic Fasciitis: A Rare and Challenging Diagnosis in a Free Clinic Setting

**DOI:** 10.7759/cureus.25668

**Published:** 2022-06-05

**Authors:** Nicholas Blackmond, Joshua Kanke, Kira Brown, Raymond Weitzman

**Affiliations:** 1 Internal Medicine, Dr. Gary Burnstein Community Health Clinic, Pontiac, USA; 2 Pharmacology, College Pharmacy, Colorado Springs, USA; 3 Internal Medicine, Ascension Providence Hospital, Birmingham, USA; 4 Rheumatology, Dr. Gary Burnstein Community Health Clinic, Pontiac, USA

**Keywords:** prednisone, methotrexate, eosinophil to lymphocyte ratio, free clinic, eosinophilic fasciitis

## Abstract

Eosinophilic fasciitis (EF) is a rare ailment that affects the immune system. Due to the rarity of this condition, there are few clear diagnostic criteria for clinicians to focus on. This may lead to significant delays in reaching a diagnosis and offering proper treatment, and patients may end up seeing multiple different specialists. This is especially true in a free clinic setting where continuity of care, follow-up, and specialist access are usually lacking. In this report, we describe a case of a 24-year-old white male who presented with sudden onset of redness, swelling, burning, and pain in the bilateral upper and lower extremities. Through arduous workup and testing, he was found to have increased eosinophils in peripheral blood, elevated levels of white blood cell count, increased C-reactive protein, and pathological changes in the tissue showing eosinophil and lymphocyte infiltration. We shed light on the relative rarity of this condition and its similar clinical characteristics to various dermatological/rheumatological disease processes. We also highlight how a free clinic can provide high-quality healthcare to bridge gaps in access to care by providing high-quality and broad specialist access while ensuring continuity of care.

## Introduction

Eosinophilic fasciitis (EF) is a rare and misunderstood condition that affects the immune system; it classically presents with acute findings that are typically accompanied by eosinophilia and includes erythema, swelling, and induration of the extremities which manifests as symmetrical involvement in the limbs or trunk [1]. Currently, there are no clear diagnostic criteria for clinicians to focus on, and hence the diagnosis is made through exclusion and close examination of the skin and histological findings are essential [1]. Due to the rarity of EF, there may be significant delays in establishing a proper diagnosis and initiating appropriate treatment, and patients often end up seeing multiple different specialists [2-7]. In 1974, Shulman first described patients who presented with diffuse fasciitis and eosinophilia and scleroderma-like hardening of the skin. Later, Rodnan et al. reported similar cases and proposed the name “eosinophilic fasciitis” due to the presence of eosinophilic infiltration and peripheral eosinophilia in the hypertrophied fascia. So far, approximately 300 patients with this condition have been reported worldwide [1,4]. The challenges associated with diagnosing and treating the condition are especially conspicuous in a free clinic setting where continuity of care, follow-up, and specialist access are usually lacking [8] and extra emphasis must be placed on proper recognition, testing, and prompt treatment in all facets of healthcare.

## Case presentation

The patient was a 24-year-old white male who presented to a free clinic in Michigan for care. He had no significant past medical history and had not consulted a physician over the last couple of years. At the time of his initial presentation in 2017, he had developed sudden-onset redness, swelling, and burning of the skin, which covered the anterior portion of both his shins and forearms. He reported that he avoided all physical exercises due to pain while walking and had been confined to his bed over the last month. He did not report any symptoms of Raynaud’s disease, difficulty in swallowing or breathing, fevers, or any recent suspicious environmental exposures such as those to rapeseed oil. Additionally, he had no family history of autoimmune diseases.

Physical exam showed indurated skin patterns from the elbows to the hands with elbow flexion contracture and decreased range of motion with elbow extension. He also had bilateral edema in the lower legs, which was indurated, erythematous, and tenderness to the calves. Flexion contraction of the knees was also observed, which had led to decreased range of motion affecting his balance. His gait pattern was stiff, and he was only able to take small, deliberate steps in a heel-strike pattern.

A blood serum test showed a high white blood cell count of 13.8 x 10^9^/L (normal range: 3.7 - 11) and high eosinophil levels at 2.6 x 10^9^/L (normal range: 0 - 0.5). The hemoglobin level and platelets were within normal ranges (Table 1).

**Table 1:** Complete blood count with differentials CBC: complete blood count; WBC: white blood cell count; RBC: red blood cell count; HGB: hemoglobin; MCV: mean corpuscular volume; ESR: erythrocyte sedimentation rate

CBC with differential	Value	Reference range
WBC	13.8 x 10^9^/L	3.7 – 11
RBC	5.06 x 10^12^/L	3.8 – 5.2
HGB	13.8 g/dL	12 – 16
Hematocrit	43.5%	35 – 46
MCV	86.0 fL	80 – 99
Platelets	438 x 10^9^/L	140 – 440
Eosinophils	2.6 x 10^9^/L	0 – 0.5
Neutrophils	7.2 x 10^9^/L	1.5 – 10.0
Lymphocytes	3.0 x 10^9^/L	1.0 – 3.5
Monocytes	0.9 x 10^9^/L	0 – 1.0
Basophils	0.1 x 10^9^/L	0 – 0.2
ESR	14 mm/hr	0 – 22

A complete metabolic panel showed a high aldolase level of 10.1 U/L (normal range: 1.2 - 7.6). The blood urea nitrogen, creatinine, glomerular filtration rate, alkaline phosphatase, and aspartate aminotransferase/alanine aminotransferase (AST/ALT) were within normal ranges (Table 2).

**Table 2:** Complete metabolic panel BUN: blood urea nitrogen; GFR: glomerular filtration rate; AST/SGOT: aspartate aminotransferase/serum glutamic-oxaloacetic transaminase; ALT/SGPT: alanine aminotransferase/serum glutamate-pyruvate transaminase

Blood test	Value	Reference range
Sodium	136 mEq/L	135 – 144
Potassium	4.1 mEq/L	3.5 – 5.3
Glucose	89 mg/dL	70 – 99
BUN	8 mg/dL	7 – 25
Creatinine	0.63 mg/dL	0.60 – 1.20
GFR	156 mL/min	>60
Alkaline phosphatase	37 U/L	27 – 120
Total bilirubin	1.1 mg/dL	0.3 – 1.0
AST/SGOT	22 U/L	13 – 39
ALT/SGPT	19 U/L	7 – 52
Total protein	5.8 g/dL	6.1 – 7.9
Aldolase	10.1 U/L	1.2 – 7.6

C-reactive protein was high at 8.2 mg/dL (normal level: <0.5), and creatine phosphokinase was low at 10 U/L (normal level: 50 - 300). Antinuclear antibody was negative based on immunofluorescence assay (IFA) using HEp-2 substrate. Rheumatoid factor, anti-RNA polymerase III antibodies, anti-centromere antibody, scleroderma antibody, and Sjogren’s antibody were all negative. Absolute immunoglobulin levels and antineutrophil cytoplasmic antibodies were normal as well (Table 3).

**Table 3:** Autoimmune blood panel, absolute values of immunoglobulin levels, antineutrophil cytoplasmic antibodies ANA: antinuclear antibody; SCL-70 antibody: scleroderma antibody; Anti-SSA/SSB antibody: Sjogren’s antibody; CPK: creatine phosphokinase; C-ANCA: antineutrophil cytoplasmic autoantibody, cytoplasmic; P-ANCA: perinuclear anti-neutrophil cytoplasmic antibodies

Blood test	Value	Reference range
ANA	Negative	-
CPK	10 U/L	50 – 300
Rheumatoid factor	<15 IU/mL	<15
Anti-RNA polymerase III antibody	Negative	-
SCL-70 antibody	Negative	-
Anti-centromere antibody	Negative	-
Anti-SSA/SSB antibody	Negative	-
Immunoglobulin G (IgG)	1385 mg/dL	791 – 1643
Immunoglobulin M (IgM)	95 mg/dL	43 – 279
IgG subclass 4	67 mg/dL	4 – 86
C-ANCA	<1:20	<1:20
P-ANCA	<1:20	<1:20

A muscle biopsy was taken from the left quadriceps muscle. The left thigh skin biopsy demonstrated chronic dermal inflammation with occasional eosinophils. The left quadriceps skeletal muscle showed patchy perimysial and endomysial chronic inflammation composed of lymphocytes and plasma cells with no eosinophils. Focal sarcolemmal staining was present on MHC immunostaining. Increased focal staining with MAC was also seen. These findings were consistent with inflammatory myopathy. The left quadricep fascia showed diffuse chronic inflammation composed of lymphocytes and plasma cells with a patchy increase in eosinophils, which were focally numerous (>40 eosinophils per high power field). Due to a laboratory processing error, we were unable to get access to the biopsy histologic photo of the eosinophilic infiltration.

The patient began receiving 60 mg of prednisone, which we tapered to 10 mg daily, and methotrexate 2.5 mg weekly as a steroid-sparing agent. He developed rebound symptoms of skin tightening and worsening pain when the prednisone was tapered below 10 mg daily. Given the nature of a free clinic and all our patients are low-income and without insurance, acquiring specific prescriptions is difficult. To obtain specific medications, the patient must apply to the pharmaceutical companies, and once approved for receiving the medication, it could take anywhere between two to four months until the medication is obtained.

We were able to stabilize the patient’s symptoms; however, due to limited improvement, adverse effects of medications, and difficulty in obtaining mycophenolate mofetil (Cellcept), the patient obtained disability insurance. After the patient's case was transferred to his new primary care physician, he was started on reslizumab, which can be used as an off-label medication for EF.

## Discussion

The etiology of EF is poorly understood. It is also difficult to diagnose due to its relative rarity and similar clinical characteristics to various dermatological/rheumatological disease processes. The pathophysiology of EF is also not completely elucidated. Firstly, there is usually an insult to fascia, commonly attributed to physical exercise (in 30-46% of patients), which leads to an autoimmune response. Other triggers include pharmacotherapy, chemical agents, infections, and hematological/autoimmune conditions [1]. This leads to a greater expression of type I collagen, fibronectin, tissue inhibitor of metalloproteinase-1, eosinophilic cation protein, and interleukin-5. Increased eosinophil migration, mast cell involvement, and other inflammatory markers are also involved in the response attributed to EF [9-10].

Most of the information on EF is found in case reports. Some selected case reports include a case of EF in which the patient’s presentation was asymmetric [2]. Another report includes dermatological signs, including peau d’orange and a groove sign [3]. Other case reports have described the typical bilateral limb edema and pathological tissue infiltration but have also emphasized that due to the rarity of the disease, delay in care can cause patient burden [5]. In one case report, 33 out of 52 patients had marked eosinophilia, elevated erythrocyte sedimentation rate, and polyclonal hypergammaglobulinemia. Additional markers such as antinuclear antibody and rheumatoid factor levels have not been found to be a consistent identifier in EF. For a definitive diagnosis, a histopathological examination from a full-thickness biopsy is required [1,2,9-14].

The study of the largest cohort of patients so far involved a retrospective epidemiologic and therapeutic analysis of about 20 million patient visits from three different medical centers. Sixty-three patients were diagnosed with EF. The patients receiving corticosteroids and methotrexate had better outcomes (64% had a complete response) compared with other treatment combinations [7]. However, some limitations of the study include its retrospective nature, not being powered enough to observe therapeutic effectiveness, and patients presenting at different points of their disease progression. In the United States, there are currently no guidelines for EF treatment, and, internationally, guidance is sparse. Lastly, in Japan, the Japanese Dermatological Association has released diagnostic criteria, severity classifications, and treatment guidelines for EF [9].

The delay in diagnosis can also lead to a significant burden on patients as described above. While it took approximately one month from presentation to diagnosis in our case, the average time to diagnosis in Wright et al.'s cohort was 11 months. One of the World Health Organization’s social determinants of health (SDH) is “access to affordable health services of decent quality” [10-12]. Free clinics are perceived to provide poor quality healthcare due to disjointed continuity of care, poor quality of health services, and limited access to specialists [8]. This case study also demonstrates how a free clinic can bring quality healthcare to bridge the SDH gaps by providing high-quality and broad specialist access while ensuring continuity of care.

## Conclusions

Apart from the unusual presentation of redness, burning, and pain in the bilateral upper and lower extremities, our patient’s hemodynamic status was stable in the setting of unprecedented high eosinophils in peripheral blood, increased white blood cell count, and elevated C-reactive protein. With early recognition and definitive diagnosis through a skin biopsy that confirmed the diagnosis of EF, we were able to stabilize and manage the patient’s symptoms. This patient had regular and frequent follow-up visits to monitor the course of his disease progression and treatment effects. Through this case report, we hope to encourage others to report similar cases and spread awareness regarding unexplained elevated eosinophils in peripheral blood along with inflammatory markers.
